# Social Experience of Captive Livingstone’s Fruit Bats (*Pteropus livingstonii*)

**DOI:** 10.3390/ani10081321

**Published:** 2020-07-30

**Authors:** Morgan J. Welch, Tessa Smith, Charlotte Hosie, Dominic Wormell, Eluned Price, Christina R. Stanley

**Affiliations:** 1 Department of Biological Sciences, University of Chester, Chester CH1 4BJ, UK; tessa.smith@chester.ac.uk (T.S.); l.hosie@chester.ac.uk (C.H.); christina.stanley@chester.ac.uk (C.R.S.); 2 Durrell Wildlife Conservation Trust, Trinity, Jersey JE3 6AP, UK; Dominic.Wormell@durrell.org (D.W.); eluned.price@durrell.org (E.P.)

**Keywords:** Livingstone’s fruit bat, social network analysis, captive welfare, binomial mixture modelling, network assortment, social roles, dominance, animal welfare, evidence based

## Abstract

**Simple Summary:**

The maintenance and stability of social experience is an especially important element of the captive welfare of zoo-housed species. The population of critically endangered Livingstone’s fruit bats (*Pteropus livingstonii*) resident at Jersey Zoo display a complex social structure of affiliative and aggressive interactions. Subgroups defined by individual characteristics contribute in different ways to this structure. Social information, illuminated through the use of social network analysis techniques, could be used in the future to promote social stability and safeguard individual welfare when making evidence-based husbandry decisions.

**Abstract:**

Social network analysis has been highlighted as a powerful tool to enhance the evidence-based management of captive-housed species through its ability to quantify the social experience of individuals. We apply this technique to explore the social structure and social roles of 50 Livingstone’s fruit bats (*Pteropus livingstonii*) housed at Jersey Zoo, Channel Islands, through the observation of associative, affiliative, and aggressive interactions over two data collection periods. We implement binomial mixture modelling and characteristic-based assortment quantification to describe the complexity and organisation of social networks, as well as a multiple regression quadratic assignment procedural (MRQAP) test to analyse the relationship between network types. We examine the effects of individual characteristics (i.e., sex, age, and dominance rank) on social role by fitting models to explain the magnitude of node metrics. Additionally, we utilize a quadratic assignment procedural (QAP) test to assess the temporal stability of social roles over two seasons. Our results indicate that *P. livingstonii* display a non-random network structure. Observed social networks are positively assorted by age, as well as dominance rank. The frequency of association between individuals correlates with a higher frequency of behavioural interactions, both affiliative and aggressive. Individual social roles remain consistent over ten months. We recommend that, to improve welfare and captive breeding success, relationships between individuals of similar ages and dominance levels should be allowed to persist in this group where possible, and separating individuals that interact frequently in an affiliative context should be avoided.

## 1. Introduction

Many species have been shown to associate non-randomly (e.g., honeybees (*Apis mellifera* L.) [1], guppies (*Poecilia reticulata*) [2], Columbian ground squirrels (*Urocitellus columbianus*) [3], African elephants (*Loxodonta africana*) [4], bottlenose dolphins (*Tursiops truncatus*) [5], Japanese macaques (*Macaca fuscata*) [6], feral goats (*Capra hircus*) [7], and yellow baboons (*Papio cynocephalus*) [8]), leading to distinct patterns of association within larger collectives at the group or population level [9]. Additional layers of social complexity arise due to differentiated social roles within groups based on sex, breeding status, morphological caste, and individual differences to name a few [10,11]. The emergent social structure of these groups is defined by the nature, quality, and patterning of constituent relationships over time, which has been suggested to influence the individual fitness of group members [12]. Additionally, social structure (as a product of behavioural interactions over time) has been shown to be linked to individual welfare in many species [13]. Consequently, changes in overall social structure or individual social role can be indicative of changes in individual health and welfare status [14].

Because of this link, many modern zoo-based animal management systems have recognized the importance of maintaining species-specific social structures when seeking to safeguard individual welfare in captivity [15]. To meet this aim, new tools for improving the subjective experience and affective states of captive animals continue to be developed [16]. Social network analysis is one such tool that has been suggested to be able to provide husbandry teams with insight into how changes in the social or physical environment, as well as changes in routine, affect the overall structure of a group and the bonds within that group [17]. Changes in social stability and individual social role can indicate fluctuations in complex affective states in an easily quantifiable way [16].

Social network theory allows for the analysis of the connection between individual constituent behaviour and the functionality of a group from an evolutionary and ecological perspective [12,14]. When applied to animal systems, social network analysis can produce a graphical representation of dyadic interactions within a group, based on adjacency matrices, which is used to identify individuals or classes of individuals (referred to as nodes) crucial to the structural integrity of the group [14,17]. This information can then inform species-specific husbandry decisions, including future population planning, institutional translocations, current enclosure changes, and group structure manipulation to maximize social stability and individual welfare [17]. However, the application of social network theory to zoo animal management is currently in its infancy.

Here, we apply social network analysis to improve the understanding of the species-specific social organisation of the Livingstone’s fruit bat (*Pteropus livingstonii*), which will aid in the evidence-based welfare assessment and captive management of this species and other closely related fruit bat species. As many fruit bat species are endemic to islands subject to extreme effects of climate change, the role of zoos and captive breeding in conservation efforts has only increased [18]. Evidence-based management of fruit bat species is currently impeded by a lack of research on their captive welfare [19]. The welfare of bats in captivity may be acutely important to the success of breeding programs, as poor welfare has been directly linked to decreased reproductive success in other social species [20,21,22]. However, there remains little research on how to best assess or improve the welfare of bat populations housed in captive environments. 

Previous studies of bat sociality in situ have employed a wide variety of network-based methodologies and have increasingly emphasized the importance of social interaction to bat population health and integrity [23]. Several species have been shown to associate non-randomly, have preferred partners, and form distinct communities that sometimes exist across several different roost sites [23]. We demonstrate here that the quantification of the social environment can be similarly applied in captive settings, through social network analysis, to maximize the evidence-based welfare management and ex situ conservation of fruit bat species through an improved understanding of captive sociality and social roles. We believe that this is the first study to implement these techniques on a zoo-housed fruit bat species, the Livingstone’s fruit bat.

The Livingstone’s fruit bat (*Pteropus livingstonii*) is native to the islands of Anjouan and Moheli of the Comoros West-Indian archipelago, off the coast of northern Madagascar [24,25]. The total wild population is thought to consist of approximately 1120 individuals [24]. The IUCN (International Union for Conservation of Nature) first listed *P. livingstonii* as endangered in 1988, and then as critically endangered in 1996 [25]. To safeguard the genetic diversity of the species, a captive breeding program was established in 1989 when the Durrell Wildlife Conservation Trust signed an agreement with the Comorian government to capture several wild individuals [24,25]. Four expeditions brought 17 individuals to Jersey Zoo, Channel Islands, by 1995 [26]. 

In 2020, the captive population of *P. livingstonii* consists of 67 individuals housed across three institutions (including one surviving member of the wild-caught group of individuals). Jersey Zoo still houses the majority of individuals, with a total population of 60 bats. The action plan formulated by the IUCN Species Survival Commission Chiroptera Specialist Group specifically recommends further research on the feeding ecology, population biology, and the social organisation of *P. livingstonii* to ensure the success of future conservation interventions [27]. Therefore, we implement social network analysis techniques on this critically endangered species to address these recommendations, as well as provide a framework as to how they might be utilized in other captive settings.

Early (unpublished) data collected by our group on the social experience of female *P. livingstonii* in captivity suggests that preferred affiliative associations exist and that these are based on kinship and age class homogeneity [28]; however, the overall social structure of a mixed sex and age group of *P. livingstonii* has not previously been elucidated. This study implements novel social network techniques to quantify the social environment experienced by individuals of this species through the analysis of observational data on proximity-based association, and affiliative and aggressive interactions, over two discrete seasons. We utilize this information to explore social structure through the quantification of levels of complexity, the relationship between spatial association and more complex types of interaction, and by the identification of trait-based assortment (individuals choosing to associate based on similarity). Additionally, we implement node metrics to determine whether individual social roles exist, and if so, whether they are predicted by the sex, age, or dominance level of individual *P. livingstonii*. The ultimate aim of this research is to demonstrate the implementation of social network analysis in a captive environment, while simultaneously aiding zoo management in making evidence-based decisions to improve individual welfare, based on an increased knowledge of social structure in this species. 

## 2. Materials and Methods

### 2.1. Ethics

Ethical approval was granted for this study by the University of Chester’s Faculty of Medicine, Dentistry and Life Sciences Research Ethics Committee on 27/3/19, reference number 1535/19/MW/BS. Use of the study population was granted in writing by the Durrell Wildlife Conservation Trust on 12/03/19.

Prior to the commencement of the study, ten days were spent learning to identify individuals and to accurately define behaviours; this also allowed the bats to become habituated to the presence of the researcher (MJW) within the enclosure. Keepers enter the enclosure on a routine basis for management purposes, so the presence of the researcher was not deemed to produce any additional stress in the study individuals. A minimum distance of two meters was maintained between the observer and the bats. Each bat had been previously implanted with a PIT tag for identification purposes. This allowed the researcher to verify individual identities during this initial phase using an RFID reader, a process that is part of their routine husbandry and does not require physical contact with the bats. All health and safety guidelines put in place by Jersey Zoo regarding entry into the enclosure and non-contact with animals were followed. 

### 2.2. Study Population

Data collection was conducted by MW at Jersey Zoo, Channel Islands, on 35 days between June and September 2019 (defined as the Summer 2019 data collection period) and on 20 days between February and March 2020 (defined as the Spring 2020 data collection period). The Spring 2020 data collection period was curtailed due to the Covid-19 pandemic. The study population consisted of 44 captive *P. livingstonii* (24 female and 20 male) during the Summer 2019 data collection and 50 *P. livingstonii* (28 female and 22 male) during the Spring 2020 data collection. Individuals were identified during both observation periods by ear notches, back patch shape, colouration, and prominent wing holes, all of which showed significant inter-individual variation and remained consistent over time. All *P. livingstonii* at Jersey Zoo were microchipped with a PIT tag at approximately eight months of age during routine zoo husbandry that could be manually read using an RFID reader to verify identification [29]. 

The age of all individuals was known at the commencement of the study as the date is recorded at birth (Supplementary Materials Tables S1 and S2). Only individuals above eight months of age at the start of data collection and that were independent from their dams were sampled in each observation period. Five individuals in the population during the Summer 2019 period and two individuals in the Spring 2020 had not yet been microchipped because they were less than eight months old and were therefore excluded from data collection. A three-letter code, based on the first letters of house names, as recorded in official zoo databases, was assigned to individuals for the purpose of data analysis and identification during the study.

**Figure A1:**
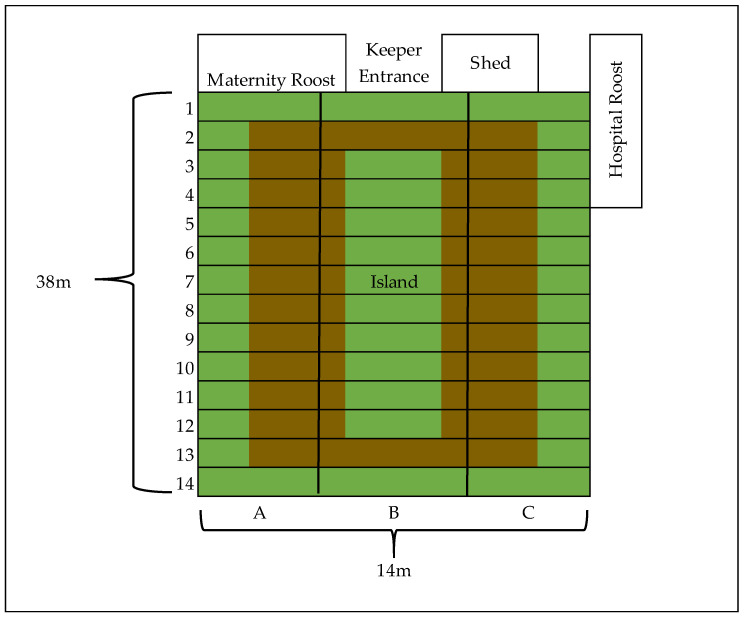
Aerial representation of the “Islands Bat Roost” at Jersey Zoo. Green sections represent accessible keeper walkways. Brown sections denote the 1.5 m deep trench dug to maximize flight paths. The enclosure was divided into 42 approximately equal sections (approximately 4.67 m wide × 2.7 m long) for data collection. These sections, bordered by pre-existing structural poles within the enclosure, were each assigned a letter A–C (west to east) and a number 1–14 (north to south).

The study population of *P. livingstonii* was housed in a heated enclosure consisting of two connected agricultural polytunnels. The enclosure is 38 m long × 16 m wide × 4 m high. A 1.5 m deep circular trench has been dug around a central raised section to increase the maximum height to 5.5 m. A shed at the north end of the enclosure was used for temporary isolation (e.g., during veterinary intervention) and a section separated from the main tunnel by a wall of mesh was used as a maternity roost. A hospital roost along the eastern wall, which housed older and injured individuals, was also separated by a mesh wall from the main tunnel (Appendix A Figure A1). Individuals housed in the maternity and hospital roosts were not included in this study because of their limited interaction with the main population and were not included in the previously stated study sizes. 

**Figure A2:**
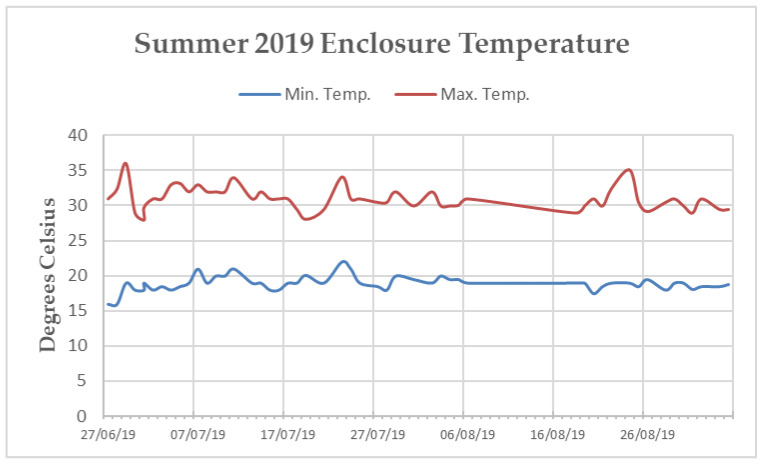
Line graph of the minimum and maximum average temperatures reported by the internal temperature monitoring system of the “Island Bat Roost” at Jersey Zoo between June and September of 2019. Dates listed are ten days apart. Tick marks along the x-axis denote one day.

**Figure A3:**
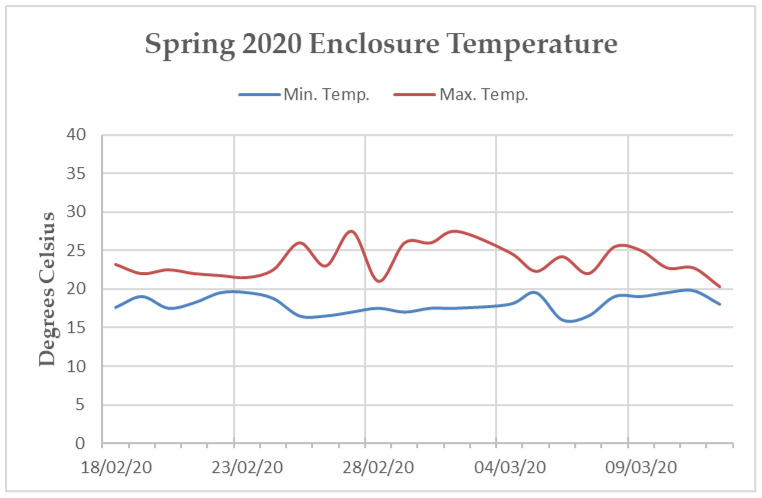
Line graph of the minimum and maximum average temperatures reported by the internal temperature monitoring system of the “Island Bat Roost” at Jersey Zoo between February and March of 2020. Dates listed are five days apart. Tick marks along the x-axis denote one day.

The main enclosure tunnel was heavily planted with soft *Ficus* sp. and *Tradescantia* sp. along the sides and bottom. Artificial turf covered the keeper walkways along the western and eastern walls, as well as the “island” (a raised section in the centre of the enclosure, surrounded by the 1.5 m deep trench). The ceiling and walls of the enclosure were covered in medium density mesh and rope [19]. Temperature within the tunnel fluctuated throughout the year, but efforts were made via the implementation of industrial fans, a mister system, a 45 kW biomass hot-air heater, and extensive insulation to maintain a minimum temperature of 16 °C and a maximum temperature of 32 °C [19]. The northern end of the enclosure sustained temperatures within this range during both data collection periods. However, the southern end remained, on average, three degrees cooler than the northern end during the Spring 2020 data collection (Appendix B Figure A2 and Figure A3). Humidity varied within the enclosure from 65% to 95%.

The study population of *P. livingstonii* shared this enclosure with a population of 15 male and female Rodrigues fruit bats (*Pteropus rodricensis*) [30]. *Pteropus rodricensis* conspecific interactions and their interactions with *P. livingstonii* individuals were not recorded as part of this study due to time constraints. Interactions with cohabitating heterospecifics could be the focus of future research, as inter-species social networks have been shown to have important implications for relevant ecological processes within populations (e.g., information transmission [31], habituation to environmental changes [32], etc.).

All bats were fed twice daily, at approximately 11:00 and 16:00. Both feeds were distributed between 65 dispenser cups, suspended from the ceiling around the perimeter of the enclosure, and a series of short lengths of Perspex gutter, affixed to the western wall. During the Summer 2019 period, feeds consisted of a Mazuri leaf-eater primate diet (Mazuri Exotic Animal Nutrition, St Louis, MO, USA), soaked in water twice a week, and all other feeds consisted of a mixture of chopped fruits and vegetables. During the Spring 2020 period, all morning feeds consisted of a Mazuri leaf-eater primate diet, and all afternoon feeds consisted of a mix of chopped fruits and vegetables. On Sundays, the fruit and vegetable mixture also contained hard-boiled eggs. Individuals that had recently undergone medical interventions or were currently nursing dependent offspring were also fed an extra portion of banana each morning.

### 2.3. Behavioural Observations

Data were collected for 5 to 6 h a day between 9:00 and 17:00 on five randomly allocated days during each week of the study periods. A total of 645 and 383 ten-minute focal observations were conducted during the Summer 2019 and Spring 2020 periods, respectively. During a brief pilot study, the enclosure was hypothetically divided into 42 approximately equal-sized sections (4.67 m wide × 2.7 m long), delineated by pre-existing columns within the enclosure (Appendix A Figure A1). A starting section (2C) was then randomly chosen before the commencement of the study period. Starting with this section, focal sampling [33] was used to record the behaviour of each individual in turn within the section, sampling all individuals within the section from north to south and then continuing in a clockwise direction to the next section. Individuals that had previously been sampled that day were not sampled again within a 24 h period. Sampling began each day in the section one clockwise in the rotation from the section that sampling had ended in the previous day.

Behaviours carried out by the focal individual at the start of the sampling period and all subsequent behavioural changes were recorded continuously during a ten-minute focal period, using the Animal Observer (version 1.0) iPad application [34]. The duration of each behaviour was recorded in seconds. Behavioural definitions were taken from the ethogram developed by Courts [26] for this species (Appendix C Table A1). For all social interactions with conspecifics, as well as the duration of the interaction, the actor and recipient’s three-letter IDs were recorded, thus conserving directionality of interactions. Heterospecifc interactions between *P. livingstonii* and *P. rodricensis* were not recorded. If the focal individual moved to a different section of the enclosure, the observer followed and continued to record data. If locomotion caused the observer to lose track of the individual, the focal observation was terminated. The focal individual’s nearest neighbour, defined as the conspecific closest to the focal individual regardless of distance [35], as well as the enclosure section that they currently occupied were recorded once per minute during the focal period. If two or more individuals were equidistant from the focal, both individuals’ IDs were recorded. Following the end of the focal observation, the RFID reader was used to manually verify the ID of the nearest neighbour or of the receiver of a dyadic behaviour if they were previously unknown. 

### 2.4. Social Network Construction

Nearest-neighbour data, obtained during focal sampling, were used to construct an association adjacency matrix for each data collection period (i.e., Summer 2019 or Spring 2020), based on the total number of scans each focal was recorded as being nearest neighbour to every other conspecific [36]. Instances where two or more individuals were recorded as the nearest neighbour were treated as two or more separate data points with the same time and date stamps. An association index was then calculated for each pair of individuals using a modified version of the Simple Ratio Index [36,37]

X/(Y_AB_ + Y_A_ + Y_B_ + X) (1)

where X represents the number of focal samples where A is the focal individual and B is their nearest neighbour, Y_A_ represents the number of focal samples where A is the focal individual and B is not the nearest neighbour, and Y_B_ represents the number of focal samples where B is the focal individual and A is not the nearest neighbour. Y_AB_, which corresponds to the number of times individuals A and B are both observed but are not nearest neighbours, always equals zero (Supplementary Materials Tables S3 and S4). This index was selected as all individuals were identifiable during data collection, but inter-individual differences in gregariousness, and therefore likelihood of being sampled, is controlled for [37]. 

Dyadic interactions were classified as either affiliative or aggressive in nature according to behavioural categories (Appendix C Table A1) described in the Courts’ [26] ethogram for *P. livingstonii*. Duration in seconds of interactions between each pair of individuals was used to populate the affiliation and aggression adjacency matrices for each data collection period. This method, instead of using the absolute number of interactions, allowed for longer and more intense interactions to be given appropriate weighting within the matrix. The Simple Ratio Index was then calculated as the association index [36]. Instead of numbers of interactions between individuals A and B, the time individuals A and B spent interacting was utilized. X corresponds to the amount of time when A was the focal individual that they interacted with B. Y_A_ represents the length of time when A was the focal but interacted with individuals other than B. Y_B_ represents the time when B was the focal individual and interacted with individuals other than A. Y_AB_ remained zero (Supplementary Materials Tables S5–S8).

The package “igraph” (version 1.2.4.2) [38] was used in the R environment (version 3.6.1) to construct three directed, weighted networks from the association, affiliation, and aggression matrices for later analysis. Graphical representations of these networks were created in the UCINET 6 network visualisation tool, NetDraw [39].

### 2.5. Social Network Analysis

#### 2.5.1. Social Complexity and Structure in *P. livingstonii*

Social complexity in animal systems can be simply defined as the number (K) and frequency of discrete relationship types within a social group [40,41]. Binomial mixture modelling of interaction data can quantify the degree of social complexity by modelling the distribution of relationship classes within a population. Because of the directional nature of the data collected here, each dyad has two association strength values (one where A is the actor and B the receiver and one where B is the actor and A is the receiver). These association strengths can be assigned to one of K unknown classes of interaction types, based on the mean strength of interaction within that class. Each class occurs at a different frequency within its network. 

Binomial mixture modelling determines how many classes (K) are supported by the data by fitting a set of models with different values for K to the distribution of association data (based on the numerator and denominator of association indices) and choosing the best one based on a specific criterion [42]. Here, we use the Integrated Completed Likelihood (ICL) instead of the Bayesian Information Criteria (BIC) [43] to identify the best model due to its high correlation between estimated complexity and actual complexity present within a system in comparison to other criteria [44]. 

The R package “VGAM” (version 1.1-3) [45] was used to first create binomial distributions of both the numerator (as a measure of observed interaction frequency between individuals) and the denominator (as a measure of sampling effort) of each association strength value while controlling for the number of directed edges within the original network. These distributions were then used to identify the model with the number of classes (K) of interactions that was best supported by the data according to the ICL [43]. The ICL was calculated as the BIC of the model +*2E*, where *E* is the entropy of the classification matrix. This method selects the best model as that in which the least amount of uncertainty as to the classification of associations exists (see Weiss et al. [44] for further explanation of this process). 

The Shannon index of entropy (H), a measurement of the richness and evenness of components (K) within a system [46], can then be applied to the parameters described through binomial mixture modelling to determine the diversity of relationships within a network, and hence produce an estimate of the level of social complexity of the system [44]. This index (H) can be expressed as:

H = −∑ *q_k_* × ln(*q_k_*) (2)

where *q_k_* corresponds to the frequency of associations of class (K) between any two individuals within the population, as described by the parameters found through binomial mixture modelling [44,46]. Though *p*-value significance-based assessment has yet to be developed for this estimation of social complexity, social systems with more relationship types (K) will have a higher Shannon index of entropy (H), and therefore will possess a higher degree of complexity. Additionally, a system will have a higher H value (and therefore display more complexity) when the frequency that interaction types (K) occur at decreases as the mean strength of association increases [44]. 

Further, once the degree of social complexity has been established within the networks, classification of how this complexity manifests in the social environment can be investigated [37]. Proximity-based association can be predictive of other, more heterogenous, types of inter-individual interactions. A multiple regression quadratic assignment procedure (MRQAP) was used to test the power of the association network to predict the affiliation, and aggression networks from both data collection periods. The MRQAP test severs the dependence between each network by randomising the residuals from the regression on each variable, in this case, node degree, within each network [47]. A thousand randomisations [47] were generated in the R statistical package “asnipe” (version 1.1.12) [48] to estimate the effect size of inter-network correlations from each observation period, and the subsequent statistical significance of those effects.

Further classification of the structure of a social group can occur through the elucidation of assorted variables [49]. Assortment refers to the tendency of individuals of like classes to interact within a group of mixed classes [50]. The test statistic for assortment (r) can range from −1 to 1, with a positive score indicating that like individuals were tied, and therefore more likely to interact, within the network. A score of ‘0’ would indicate random mixing between the classes. This metric can be useful for predicting how individuals will form relationships within a group, and for identifying bonds that may be more important to the welfare of the individual [51]. Zoo management can utilize this type of information to prioritize housing individuals in groups with a demographic structure that is reflective of the level of assortment observed within a larger population, therefore preserving social structuring that may impact welfare.

The R statistical package “assortnet” (version 0.12) [52] was used to calculate the assortment of each network from each observation period based on sex, age, and dominance (as represented by David’s score, see next section for calculation). To test for significance, 1000 random node label permutations of each network were generated using the R statistical package “sna” (version 2.4) [53,54]. The r value of each node variable was then extracted from the random networks, stored, and compared to the r value of the corresponding observed network to calculate statistical significance of the observed class-based assortment [55]. 

#### 2.5.2. Dominance in *P. livingstonii*

Individual dominance rank, as resulting from agonistic interactions with conspecifics, has been shown to influence the social structure of groups [56]. Before factors affecting social role could be analysed, the dominance hierarchy of *P. livingstonii* had to be estimated and dominance scores assigned to individuals. David’s score, a metric that calculates the dominance rank of individuals in a group based on the outcomes of aggressive interactions, as well as the relative ranking of partnered conspecifics [57], was chosen to estimate the dominance hierarchy for captive *P. livingstonii,* based on the low value of latent steepness of the linear hierarchies, as well as the format of data collection [58]. 

The R statistical package “EloRating” (version 0.46.10) [59] was used to assign David’s scores to individuals for each data collection period based on respective aggression data. The distribution of David’s scores was assessed through a Shapiro–Wilk test and density plot. A linear model (with Gaussian error structure as the data were found to be normally distributed) was then used to determine the effect of the predictors age and sex on dominance rank [55]. The lowest Akaike information criterion (AIC) score, an estimation of a model’s fit and generalisability to the data set in question based on the model’s maximum log-likelihood, was used to indicate the best model. The best model had the lowest AIC score and was not within two delta AIC of the next model. If no model including at least one of these predictors met these criteria, then no factors were deemed to be predictive of observed David’s scores [60]. The best model was then used to determine the effect size estimates, standard error, and statistical significance of each demographic variable within the model on dominance rank [60]. The fit of the model was assessed by the examination of residuals versus fitted values and Q-Q plots [43].

#### 2.5.3. Social Roles of *P. livingstonii*

Node- or individual-based metrics, unlike network-level metrics, apply only to the individual and are entirely dependent on the scores of all other individuals within the network [61]. These types of scores define the nature of individual social roles within the larger social experience. To identify the influence of class membership on social roles, we examined the relationship between sex, age, and dominance (as represented by David’s scores) on three important node metrics; betweenness centrality, closeness centrality, and weighted degree.

Betweenness centrality is defined as the number of shortest paths that pass through a node, where the shortest path refers to the lowest number of edges between two nodes [62]. More practically, individuals with high betweenness have been shown to play key roles in social transmission between disparate subgroups within a network [9]. Removal of these individuals from a network often results in the fragmentation or complete dissolution of the group [63]. Closeness centrality refers to the connectivity of a node within its network, according to the number and strength of its edge connections, as well as the connectivity of the nodes to which it is directly tied [64]. Unlike betweenness centrality, the calculation of this score accounts for edge direction, making it highly useful in the analysis of weighted networks [65]. Individuals with high closeness centrality are thought to have a larger relative ‘influence’ over the decisions of the group [9]. Weighted degree describes the number of edges that a node is connected to, while accounting for the value of those edges [66]. This score generally describes the immediate strength of an individual’s relationships with other directly connected nodes within the network and does not account for directionality [61].

The R statistical package “sna” (version 2.4) [53] was used to calculate the weighted degree and “igraph” (version 1.2.4.2) [38] was used to calculate the normalized betweenness centrality and normalized closeness centrality of each *P. livingstonii* within each network (with normalized scores controlling for differences in network sizes between the two seasons due to demographic changes in the population). Closeness centrality measures were normalized by multiplying raw scores by *n*-1, where *n* represents the number of nodes within each network. Betweenness centrality measures were normalized through the equation

*Bnorm* = 2*B*/(*n*^2^ − 3*n* + 2) (3)

where *Bnorm* is normalized betweenness centrality, *B* is the raw betweenness score, and *n* is the number of nodes within each network. All individuals were given a different score for each of the three network types from the separate data collection periods, for a total of six node metrics per individual. A Shapiro–Wilk test, along with the visual inspection of density plots, was used to assess the distribution of weighted degree, closeness centrality, and betweenness centrality scores.

To investigate the effects of class membership on node metrics and subsequently social role, a linear model (with Gaussian error structure, as normalized node metrics were normally distributed) including the predictors sex, age, and dominance rank (as represented by David’s scores) was implemented for each node metric from each network, giving a total of 18 models [55]. The model with the lowest AIC score that was not within two delta AIC of any other model was used to determine which, if any, of these predictors provided the best fitting model for each set of node metrics [60]. The best fitting models were then used to determine effect size estimates [60,61]. As previously described, the fit of the model was assessed by the examination of residuals versus fitted values and Q-Q plots [43].

To account for the dependent nature of network-based metrics, each network was then permuted 1000 times using node label permutations in the R statistical package “sna” (version 2.4) [53,54]. These randomisations kept the total time individuals spent as the actor and receiver of sociality the same by permuting data only within rows and columns of the SRI matrices [54]. The normalized betweenness centrality, normalized closeness centrality, and weighted degree for each node within the randomized networks was then extracted and stored. The best fitting model of the effect of the predictors on node metrics from observed networks was then applied to each randomized network. The coefficients from the model applied to the observed networks were then compared to the distribution of coefficients extracted from models based on 1000 random networks to determine the statistical significance, if any, of the observed effects. This method allowed for linear modelling to be applied to network-based data [37].

To test for temporal stability of social roles, as described by network position over time, normalized node metrics calculated in the R statistical packages “sna” (version 2.4) [53] and “igraph” (version 1.2.4.2) [38] for each network were aggregated into three, network type-based arrays—one comparing metrics from both association networks, one comparing metrics from both affiliation networks, and one of metrics from both aggression networks. Individuals that were not observed in both data collection periods were removed from these arrays using “tidyverse” (version 1.3.0) [67] for a total of 42 conserved nodes. A quadratic assignment procedure (QAP) test [68] was then run on each array to test how node metrics calculated in the first data collection period predicted scores calculated in the second one. If social role is consistent over time, we would expect node metrics from the Summer 2019 data collection to positively predict node metrics from the Spring 2020 data collection [68]. 

## 3. Results

### 3.1. Data Collection Summary

The mean duration of total focal observations of each individual during the Summer 2019 data collection period was 151.28 min with a standard deviation of 0.922 min. During the Spring 2020 data collection period, the mean duration of total focal observations for each individual was 82.74 min with a standard deviation of 6.82 min. This data collection period was significantly curtailed by the Covid-19 pandemic.

### 3.2. Social Complexity and Structure

#### 3.2.1. Network Complexity

Through binomial mixture modelling [44], each network type was found to support either three or four categories of interaction (K), occurring at different frequencies within each network (Table 1), and to display varying levels of social complexity (H) [40]. The interaction type of least intensity (i.e., the type that had the lowest mean strength of association) occurred at the highest frequency within each network, suggesting that individuals had associations of low strength with many conspecifics, but only interacted more intensely with a small number of other individuals. 

Both the association and aggression networks decreased in their estimated level of complexity from the Summer 2019 data collection period to the Spring 2020 period, whereas the affiliation network increased in complexity. The association network from the Summer 2019 data collection period displayed the highest degree of social complexity with the highest Shannon entropy index (H = 1.214; Table 1) and the aggression network from the Spring 2020 data collection period displayed the least complex social structure, with the lowest Shannon index (H = 0.507; Table 1). It is important to note however, that models selected by ICL are often underestimations of social complexity at lower sampling levels [44]. Because of this, the decrease in H in the association and aggression networks between the Summer 2019 and the Spring 2020 data collection periods may be influenced by the lower number of observations conducted in Spring 2020.

#### 3.2.2. Relationships between Network Types

**Figure 1:**
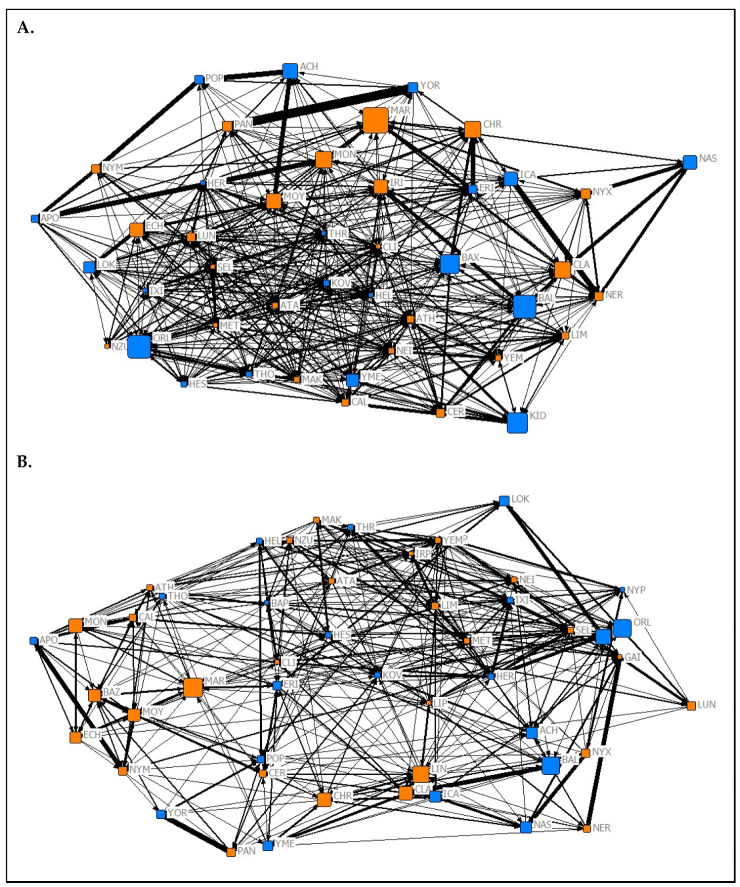
Graphical representations of the observed association networks from (**A**) Summer 2019 and (**B**) Spring 2020 of captive *P. livingstonii* created in the UCINET 6 visualisation tool NetDraw [39]. Nodes are labelled with their assigned three letter codes. Blue nodes denote male individuals, and orange denote females. The size of each node corresponds to the age in years of the individual during respective data collection periods with older individuals being represented by larger nodes. The width of each tie increases with the weight of the edge. Non-metric geo-distances with node repulsion were used to create the layout, meaning nodes with more similar ties are closer together and nodes that are more central to the network are placed closer to the centre of the graph.

The results of the MRQAP test suggest that the association network from the Summer 2019 data collection period (Figure 1A) predicted both the affiliation (*t* = 24.08, *p* < 0.05) and aggression (*t* = 10.38, *p* < 0.05) networks from the same period. The association network from the Spring 2020 data collection period (Figure 1B) also significantly predicted both the affiliation (*t* = 26.6, *p* < 0.05) and aggression (*t* = 3.35, *p* = 0.009) networks from its respective data collection period. In both data collection periods, association tie strength was more likely to predict affiliative ties rather than aggressive ones. 

#### 3.2.3. Network Assortment

All statistically significant network assortment was positive (Table 2), meaning individuals chose to interact with conspecifics of the same class more often than would be expected if interactions were random [50]. No network was assorted by sex, suggesting that individual sex does not play a role in interaction preference in any network type. The significant assorted variables for each network type varied between the Summer 2019 and Spring 2020 data collection periods. However, every network was assorted positively by either age or dominance, except for the Summer 2019 affiliation network, which did not display any statistically significant variable assortment. Possible causes of these seasonal deviations will be explored in further sections. 

### 3.3. Social Roles

#### 3.3.1. Dominance Hierarchy Analysis

The dominance hierarchies, as determined by David’s scores, from each data collection period were not linear (Summer 2019: h = 0.017, *p* > 0.05; Spring 2020: h = 0.006, *p* > 0.05), suggesting that something other than the outcome of aggressive interactions may affect dominance in captive *P. livingstonii.* This result could also indicate that the hierarchy is circular and therefore more like an aggression network than a hierarchy. These results could also be an artifact of the need for more long-term data to reveal structure (whether this is linear or not) [69,70]. The model that best predicted (i.e., had the lowest AIC score) the observed dominance rankings consisted singularly of age for both data collection periods. The effect of age on dominance was 1.034 (*p* = 0.017, SE = 0.415) for the Summer 2019 period and 0.317 (*p* = 0.008, SE = 0.116) for the Spring 2020 data collection period. This indicates that older individuals were more likely to be dominant in both time periods, regardless of sex.

#### 3.3.2. Class Membership and Social Roles

The best model for predictive variables identified for each node metric differed between the Summer 2019 and Spring 2020 networks of the same type (Table 3), suggesting that class membership may influence social roles differently during different times of the year. Not every best model for node metrics was found to give statistically significant predictors after permutation testing, suggesting that class membership alone does not assign social role in *P. livingstonii*. Other variables, such as individual personality, may have some influence and should be explored in further studies. All predictors found to be statistically significant occurred in models predicting aggression network node metrics. Specifically, these results suggest that during the Summer 2019 data collection period, more dominant individuals had a higher degree of closeness centrality and males had higher weighted degrees than females. During the Spring 2020 data collection period, males also appear to have had a higher degree of closeness centrality within the aggression network.

#### 3.3.3. Temporal Stability of Social Roles

**Figure 2:**
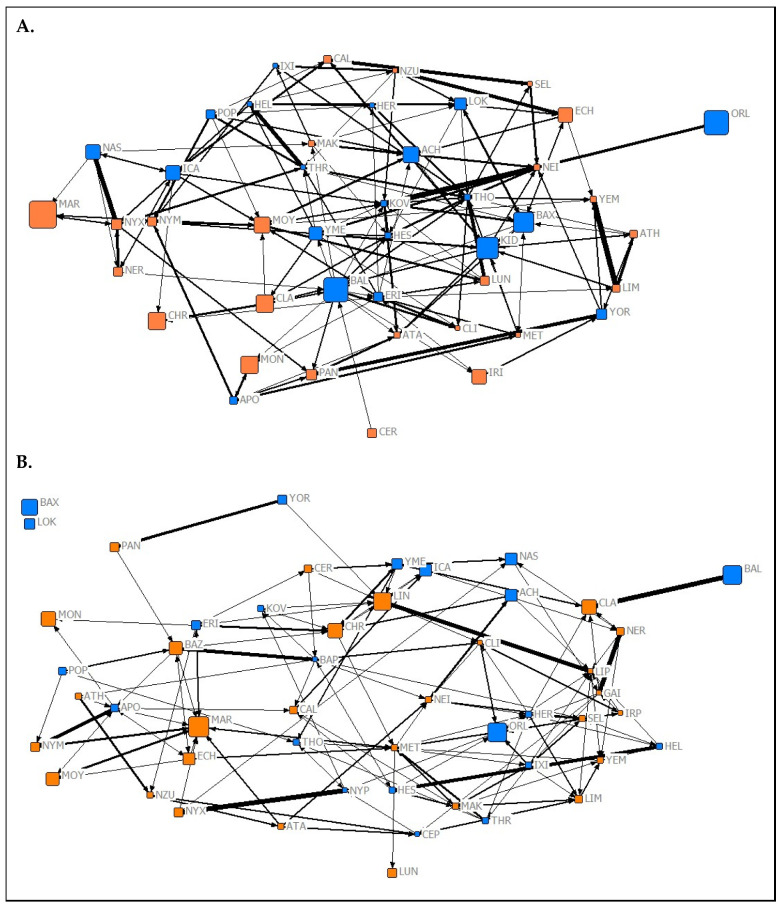
Graphical representations of the observed affiliation networks from (**A**) Summer 2019 and (**B**) Spring 2020 of captive *P. livingstonii*. Nodes are labelled with their assigned three letter codes. Blue nodes denote male individuals, and orange denote females. The size of each node corresponds to the age in years of the individual during respective data collection periods with older individuals being represented by a larger node. The width of each tie increases with the weight of the edge. Non-metric geo-distances with node repulsion were used to create the layout, meaning nodes with more similar ties are closer together and nodes that are more central to the network are placed closer to the centre of the graph.

**Figure 3:**
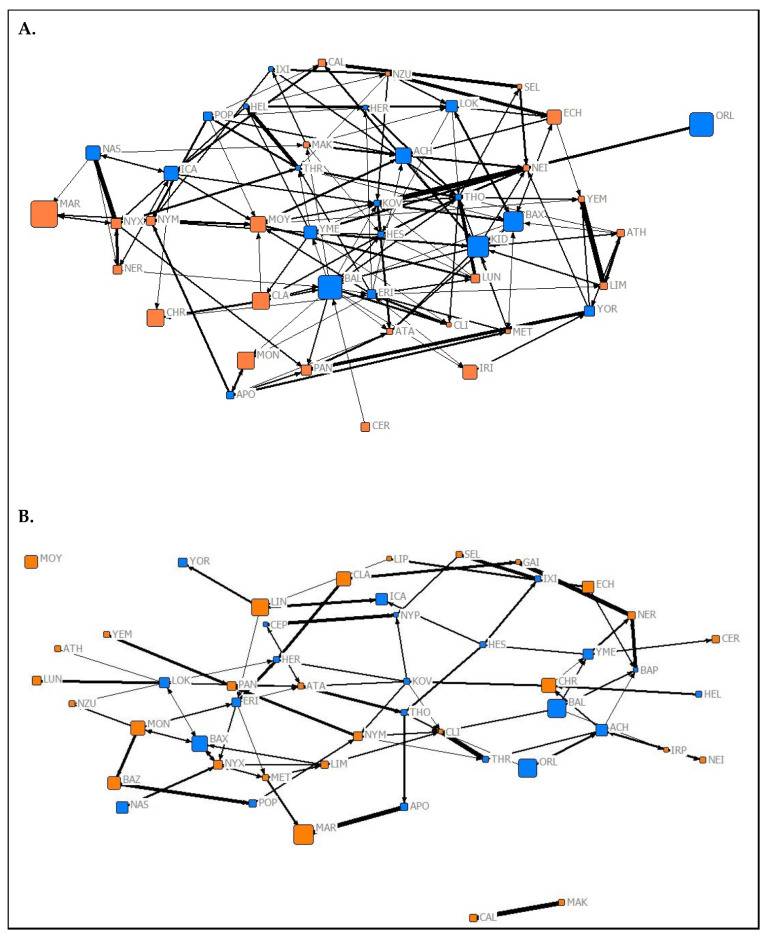
Graphical representations of the observed aggression networks from (**A**) Summer 2019 and (**B**) Spring 2020 of captive *P. livingstonii*. Nodes are labelled with their assigned three letter codes. Blue nodes denote male individuals, and orange denote females. The size of each node corresponds to the age in years of the individual during respective data collection periods with older individuals represented by larger nodes. The width of each tie increases with the weight of the edge. Non-metric geo-distances with node repulsion were used to create the layout, meaning nodes with more similar ties are closer together and nodes that are more central to the network are placed closer to the centre of the graph.

The results of the QAP test used to investigate the temporal stability of social roles showed that normalized node metrics extracted from each 2019 network were positively predictive of normalized node metrics extracted from corresponding networks from Spring 2020 (association network (Figure 1) node metrics: r = 0.9704, *p* < 0.05; affiliation network (Figure 2) node metrics: r = 0.489, *p* = 0.028; aggression network (Figure 3) node metrics: r = 0.337, *p* = 0.024). These findings suggest that the social roles of *P. livingstonii* in captivity can show stability over different seasons, with association-based roles being the most highly conserved, as indicated by higher r values.

## 4. Discussion

This study has demonstrated how novel social network analysis-based techniques can be implemented to study the sociality of captive populations. Our results can now inform the management of a critically endangered species to safeguard individual welfare through the preservation of crucial social relationships and underpinning structure. Captive *P. livingstonii* display a variety of conspecific relationships and substantial social complexity. Different types of social relationships in other species (as categorized by strength and frequency of interaction) have been shown to provide varied ecological and emotional benefits within social systems [71,72]. The composition of three or four interaction types (K) (based on mean strength of association) present within this population at increasing frequency within each network may present *P. livingstonii* individuals with a gradation of relationship strengths, each with potential fitness benefits. 

Analysis of the level of crossover between types of social interaction was carried out through the implementation of an MRQAP test [47] to quantify the predictive power of affiliative and aggressive networks on proximity-based association. The results of this test showed that the association network was significantly predicted by both the affiliation and aggression networks from both data collection periods. This suggests that *P. livingstonii* interact more frequently, both affiliatively and aggressively, with conspecifics with whom they also spend more time in close proximity. However, both association networks were more highly predicted by their corresponding affiliation networks rather than their aggression networks. From a management perspective, this indicates that frequent spatial association can be interpreted as evidence of social bonds between individuals.

To further explore the underpinning variables of the observed social complexity, the dominance hierarchy of this population was assessed through the calculation of individual David’s scores. A basic examination of predictors of these scores through linear modelling revealed that age alone was found to affect dominance rank in both data collection periods. One possible explanation for the exclusion of sex as an influential factor, as would be initially assumed from previous research on harem structure in this and other related species in the wild [73], is that size has been shown to positively correlate with age in other fruit bat species [74]. Older individuals may be larger, and therefore more likely to win agonistic interactions regardless of sex [74,75]. As David’s score is based on the outcome of aggressive encounters, it would follow that older, larger individuals may be more dominant in this population. 

To further quantify the social organisation of *P. livingstonii*, the network-based assortment of variables describing individual characteristics (i.e., sex, age, and dominance rank) was calculated. No network from either data collection period was found to be significantly assorted by sex, indicating that individuals interacted with conspecifics of the same and opposite sex at relatively similar rates. This highlights the need for future research on preferential inter- and intra-sexual relationships in captivity, as previous research on the social organisation of *P. livingstonii* suggested that, because of harem-based reproduction and male resource guarding, male to male interactions were mostly aggressive in nature, and male to female interactions were mostly affiliative [73,74,75]. The lack of obvious harem structuring in this population may be due to a number of factors presented by a captive environment, including but not limited to the abundant nature of resources or the limited spatial choices available to individuals. This points to a need for the exploration of territoriality and captive space use in this species. 

Another notable social pattern present in this population identified during assortment analysis was that all statistically significant assortment was positive, suggesting that individuals have a higher degree of preference for interaction with members of like classes [50]. The aggression network from the Summer 2019 data collection period and the association network from the Spring 2020 data collection were both positively assorted by age. As well as having a similar age in years, these individuals often share a life-stage. This means that, perhaps more than age alone, *P. livingstonii* chooses in captivity to associate with conspecifics who share similar ecological goals such as social requirements, reproductive or energetic status [76]. Group cohesion in other social species (e.g., yellow-bellied marmots (*Marmota flaviventris*) [77], Merino sheep (*Ovis ares*) [78], Columbian ground squirrels (*Spermophilus columbianus*) [3], etc.) has also been shown to improve when individuals have fewer conflicting interests, so individuals of similar biological and social requirements often associate at a higher frequency [79].

In addition to age, two out of three networks (association and aggression for Summer 2019; affiliation and aggression for Spring 2020) from each data collection period were also positively assorted by dominance, as represented by David’s scores. As the calculation of David’s score is based on the outcome of agonistic interactions, it follows logically that aggression networks would be positively assorted by dominance. The positive assortment of the association network from Summer 2019 and the affiliation network from Spring 2020 suggest that different seasons may present social environments where interaction preference changes. One possible factor influencing this change may be the reproductive cycle of *P. livingstonii*. In captivity, the mating and birthing seasons are somewhat longer than what has been observed in the wild [80]. It has been estimated that parturition takes place in captivity during the warmer months from March to September, meaning that mating (although witnessed infrequently) takes place from November to March. 

Hence, it could be that Summer 2019 dominance rank-based assortment within the association network is an artifact of the individual’s choice to interact with groups that support the ecological and social needs presented by parturition and caring for young. Individuals may be more likely to thrive during this time of year if they more frequently interact with conspecifics of a similar dominance level [79]. The Spring 2020 data collection took place during the end of the *P. livingstonii* mating season. For this reason, the positive dominance-based assortment of the affiliation network from this period could be indicative of mate choice. Perhaps *P. livingstonii* preferentially choose mates with a similar, or higher dominance ranking as an indicator of positive fitness. The maximum enclosure temperature during this period was also notably lower than in the summer data collection period (Appendix B Figure A2 and Figure A3). Perhaps the lack of dominance-based assortment in the association network from this period is a result of individuals roosting in closer proximity to heating units along the northern wall of the enclosure. When space is limited in captivity, social choice is also constrained [81]. However, the interpretation of these results should be regarded tentatively, as these results may simply be an artifact of reduced data collection in the Spring 2020, due to the Covid-19 pandemic. For this reason, future exploration of variable-based assortment through further data collection of other seasons should be prioritized.

Once the overall complexity and assortment of the social structure of captive *P. livingstonii* were described, the social roles of classes of *P. livingstonii* were approximated through the estimation of the effects of predictors describing individual characteristics (i.e., age, sex, and dominance rank) on node metrics. Not every best fitting model identified a statistically significant effect, suggesting that something other than the selected characteristics alone may influence social roles. We suggest that individual differences and personality type should be investigated as a potentially significant factor in social role emergence [82,83,84]. Only models applied to node metrics characterising the aggression networks from both data collection periods were found to include statistically significant predictors. During the Summer 2019 period, more dominant individuals had a higher aggression-based closeness centrality and males had a higher weighted degree. This indicates that dominant individuals held higher strength aggression-based connections [9] and males generally had a higher number of aggression-based connections with conspecifics than did females. This is not to suggest that dominant males were the most aggressive during this period, but rather that dominant individuals (of either sex) had more intense aggressive interactions and that males were part of more aggressive interactions regardless of directionality. 

During the Spring 2020 data collection period, males had a higher aggression network-based closeness centrality than females. In contrast with the Summer 2019 period, this indicates that males held higher-intensity agonistic connections with conspecifics. This suggests that social roles based on aggressive interactions could be predicted, to different extents, by the characteristics identified here (i.e., sex and dominance), but also that the social niche of subgroups could change depending on the ecological needs of the group during different seasons.

Though the relationship between descriptive factors (i.e., age, sex, and dominance rank) and social role await further exploration, this study has also demonstrated that individual social roles can remain temporally stable. Through the implementation of a QAP test [68], individual normalized node metrics from the Summer 2019 data collection were found to be significantly predictive of node metrics from the Spring 2020 data collection in all three network types. Association network-based roles (as characterized by node metrics) showed the highest degree of temporal fidelity. This suggests that, though the variables most predictive of social role change over different seasons, individuals retain socially important standing within the group. 

## 5. Conclusions

Through the implementation of social network analysis on the behaviour of the population of *P. livingstonii* housed at Jersey Zoo, we have demonstrated the efficacy of novel methods to quantify factors affecting captive social structure in a highly social mammal. We have quantified observed social complexity through the modelling of categories of interaction based on strength and frequency, as well as through analysis of variable assortment. Additionally, we have started to unravel the ecological basis for social roles, as defined by node metrics, in this species. 

Relationship strength appears to be negatively correlated with the frequency at which that type is displayed within the population. *P. livingstonii* also appear to positively assort based on age and dominance level, with dominance being highly predicted by the age of the individual regardless of sex. Social roles may show consistency over time (shown here over a period of ten months), with variables describing the individual characteristics influencing social role assignment changing over the seasons. 

Based on the findings of this analysis, we suggest that captive management of *P. livingstonii* should allow for relationships between individuals of similar ages and dominance levels to persist where possible, and separating individuals that interact frequently in an affiliative context should be avoided. Maintaining social bonds could be extremely important for the efficiency of this captive breeding program, as social bonds have been found to play a key role in the reproductive success of other social species such as Assamese macaques (*Macaca assamensis*) [85], feral horses (*Equus caballus*) [86], and chacma baboons (*Papio ursinus*) [22] to name a few. Further, the influence of individual differences and personality on social roles, in addition to the temporal influences on social roles in this species, could be further examined as a direction for future research.

Our results have added to the growing body of literature highlighting the key role of social network analysis in evidence-based management, in particular due to the method’s power to quantify individual social experiences and therefore to safeguard individual welfare. 

## Figures and Tables

**Table 1:** This table shows the K types of interactions supported by binomial mixture modelling within each network, the mean strength of each type of interaction, the frequency of each type of interaction, and the Shannon index of entropy for each network.

Network Complexity					
Network Type	Data Collection Period	Types (K)	Mean Strength	Frequency	S Index (H)
Association	Summer 2019	4	0.001	0.772	1.214
			0.028	0.166	
			0.082	0.042	
			0.213	0.019	
	Spring 2020	3	0.000	0.861	0.689
			0.062	0.132	
			0.369	0.008	
Affiliation	Summer 2019	3	0.000	0.919	0.686
			0.108	0.058	
			0.341	0.023	
	Spring 2020	3	0.000	0.939	0.73
			0.083	0.055	
			0.643	0.006	
Aggression	Summer 2019	4	0.000	0.916	0.973
			0.036	0.042	
			0.206	0.03	
			0.381	0.012	
	Spring 2020	3	0.000	0.962	0.507
			0.129	0.025	
			1.000	0.013	

**Table 2:** This table shows the factors (age, sex, or dominance) predicting assortment, the effect size estimate of that variable and the statistical significance of that result. Statistically significant results are highlighted in bold.

Network Assortment				
Network Type	Data Collection Period	Assorted Variable	Effect Size(r)	*p*-Value
Association	**Summer 2019**	**Dominance**	**0.139**	**0.049**
	**Spring 2020**	**Age**	**0.291**	**0.002**
Affiliation	Summer 2019	N/A	N/A	N/A
	**Spring 2020**	**Dominance**	**0.332**	**0.025**
Aggression	**Summer 2019**	**Age**	**0.208**	**0.043**
		**Dominance**	**0.343**	**0.002**
	**Spring 2020**	**Dominance**	**0.237**	**0.037**

**Table 3:** This table shows the best models for predictor variables (age, sex, and dominance) on node metrics (betweenness centrality, closeness centrality, and weighted degree) for each network, as well as the effect size estimate of each variable within that model, the standard error of those effects, and the statistical significance after permutation testing. Statistically significant effects are highlighted in bold. Effect sizes are given for males relative to females where sex was a factor in the best model.

Node Metrics and Class Membership						
Network Type	Data Collection Period	Node Metric	Factors Included in Best Model	Effect Size	Standard Error	*p*-Value
Association	Summer 2019	Betweenness Centrality	Dominance	−0.0007	0.0003	0.52
	Spring 2020	Betweenness Centrality	None	N/A	N/A	N/A
	Summer 2019	Closeness Centrality	None	N/A	N/A	N/A
	Spring 2020	Closeness Centrality	Age	−0.192	0.076	0.698
	Summer 2019	Weighted Degree	None	N/A	N/A	N/A
	Spring 2020	Weighted Degree	None	N/A	N/A	N/A
Affiliation	Summer 2019	Betweenness Centrality	Dominance	−0.003	0.0007	0.514
	Spring 2020	Betweenness Centrality	None	N/A	N/A	N/A
	Summer 2019	Closeness Centrality	Dominance	−0.023	0.009	1
	Spring 2020	Closeness Centrality	Sex	Male = 0.042	0.023	1
			Age	−0.004	0.002	1
	Summer 2019	Weighted Degree	Sex	Male= −0.249	0.125	0.968
			Dominance	−0.008	0.004	0.945
	Spring 2020	Weighted Degree	None	N/A	N/A	N/A
Aggression	Summer 2019	Betweenness Centrality	Sex	Male = 0.057	0.024	0.5
			Dominance	0.003	0.0008	0.509
	Spring 2020	Betweenness Centrality	Sex	Male = 0.0009	0.0004	0.498
	**Summer 2019**	**Closeness Centrality**	**Dominance**	**0.024**	**0.013**	***p* < 0.05**
	**Spring 2020**	**Closeness Centrality**	**Sex**	**Male = 0.028**	**0.017**	***p* < 0.05**
	**Summer 2019**	**Weighted Degree**	**Sex**	**Male = 0.31**	**0.14**	**0.004**
			Dominance	−0.025	0.005	1
	Spring 2020	Weighted Degree	Dominance	−0.037	0.014	0.997

## Supplementary Materials

The following are available online at https://www.mdpi.com/2076-2615/10/8/1321/s1, Table S1: Summer 2019 Individual Attributes, Table S2: Spring 2020 Individual Attributes, Table S3: Summer 2019 Association Matrix, Table S4: Spring 2020 Association Matrix, Table S5: Summer 2019 Affiliation Matrix, Table S6: Summer 2019 Aggression Matrix, Table S7: Spring 2020 Affiliation Matrix, Table S8: Spring 2020 Aggression Matrix.

## Appendix C

**Table A1:** Ethogram of aggressive and affiliative Livingstone’s fruit bat behaviours used for data collection, as described by Courts [26].

Ethogram of Dyadic LFB Behaviours		
Category	Behaviour	Definition
Aggression	open mouth	mouth open and directed at another individual in close proximity
	food steal	one individual takes food from another with force
	lunge	one individual lunges (either whole body or head) toward another
	bite	one individual’s mouth makes forceful contact with any part of another (usually the head/neck) during an aggressive encounter
	thumb jab	the hook of the thumb is jabbed at another individual during an aggression encounter
	wrestle	intense form of aggression involving biting, thumb jabbing, and attempts at connecting blows, one or both bats may lose their grip on the mesh, accompanied by vocalisations
	double thumb jab/shove	both thumb hooks make contact with another individual during an aggressive encounter
	chase	one individual rapidly pursues another
	displace	an individual’s approach along a path causes another individual to leave the area
	flight-displaced by	one individual flies away from a location in order to be out of the path of another approaching individual
	mesh movement-avoid	one individual moves horizontally along the mesh to get out of the way of or move away from another individual
	climb-avoid	one individual moves vertically along mesh or substrate to get out of the way of or move away from another individual
	vocal-male cackle	made by males during an aggressive encounter
	vocal-call	a two tone sound used predominantly by males in territory defence, directed at an intruder, wings are often spread, frequency of calls varies with dominance level, females can produce a quieter version
	vocal-female cackle	made by females to ward off males, much louder than chatter or male cackle
	wing spread-social wing flick	one or both wings vigorously flapped toward another individual and then withdrawn
	wing spread-shaking	wings shaken repeatedly at another individual or threatening object/hazard, can be both together or alternating or with whole body movements
	wing spread-marching	wings move back and forth alternately toward another individual or a threatening object/hazard
	wing spread-rotating	whole body and wings moving 45 degrees either side of a threatening subject
	wing clap	both wings rapidly drawn together in direction of another individual, making a clapping sound as they meet
	wing ruffle	partially folded wings drawn together in direction of another individual, making a ruffling sound as they meet
Affiliation	roost in pair	hanging by hind limb(s), wings wrapped around ventral region or wings of other individual, muzzle usually tucked into chest, eyes closed, no ear movements
	allogrooming-mutual	two animals simultaneously groom each other with licking, biting, or scratching of similar intensity
	allogrooming-nonmutual	one animal within a pair grooms the other by licking, biting, or scratching with no reciprocation
	play	prolonged gentle wrestling, holding, mouthing, biting, and genital sniffing and grooming with no vocalisations, may lead to mounting
	food share	involves one individual vigorously licking the mouth of another while they chew, the feeding individual usually holds the other behind the head or both individuals hold each other
	follow	one individual follows another, not the same as chase as both individuals often stay together once movement has ceased
	approach without displacement	one individual approaches another by climbing or flying without displacement
	touch nose	two individuals make physical contact with their noses
	body/genital sniff	one individual sniffs the body, genitals, or air around another individual
	pre-copulation	involves sniffing, genital allogrooming, mutual jabbing and light wrestling
	copulatory mount	male with an erect penis mounts a female, male grips female from behind, male restrains females forelimbs with his own and holds the scruff of her neck in his mouth
	air sniff	an individual smells the air around another by directing its head and nose
	hook	one individual hooks onto or touches another with its thumbs down, more gentle than thumb jab
	intervene in aggression	a third individual attempts to break up a bout of wrestling or fighting between two other individuals, this can halt the fight, cause one or both animals to move off, or initiate aggression directed at the third party
	lean towards	movement of body only towards another individual when in the hanging position
